# Anticancer Potential of Lipophilic Constituents of Eleven Shellfish Species Commonly Consumed in Korea

**DOI:** 10.3390/antiox10101629

**Published:** 2021-10-15

**Authors:** Juhyun Shin, Min-Ho Song, Ji-Woo Yu, Eun-Young Ko, Xiaomin Shang, Jae-Wook Oh, Young-Soo Keum, Ramesh Kumar Saini

**Affiliations:** 1 Department of Stem Cell and Regenerative Biotechnology, Konkuk University, Seoul 143-701, Korea; junejhs@konkuk.ac.kr (J.S.); ohjw@konkuk.ac.kr (J.-W.O.); 2 Department of Crop Science, Konkuk University, Seoul 143-701, Korea; hlhkkl@konkuk.ac.kr (M.-H.S.); wooody96@konkuk.ac.kr (J.-W.Y.); rational@konkuk.ac.kr (Y.-S.K.); 3 Department of Food Science and Biotechnology of Animal Resources, Konkuk University, Seoul 143-701, Korea; key523@konkuk.ac.kr; 4 Jilin Provincial Key Laboratory of Nutrition and Functional Food, Jilin University, Changchun 130062, China; xmshang@jlu.edu.cn

**Keywords:** cholesterol, phytosterols, omega-3 fatty acids, tocopherol, mollusks, crustaceans, DPPH^•^ scavenging activities, ABTS^+•^ scavenging activities, glioblastoma multiforme (T98G), lung carcinoma (A549)

## Abstract

The present study was aimed to investigate the composition and contents and the major lipophilic compounds, including the sterols, fatty acids, and tocols of shellfish species. Moreover, to explore the antitumor activity of these lipophilic constituents, their cytotoxicity potentials were determined against five different human cancer cells, including colon carcinoma (HCT116), epithelial melanoma (A2058), glioblastoma multiforme (T98G), lung carcinoma (A549), and adenocarcinoma (HeLa). The results show a significant variation in the contents and composition of lipophilic constituents among the studied species. The highest omega-3 (n-3) polyunsaturated fatty acids (PUFAs) were recorded from arrow squid and pacific oysters, accounting for 53.2% and 53.0% of their total fatty acids, respectively. However, the highest cholesterol content was also recorded in arrow squid (154.4 mg/100 g; 92.6% of total sterols). In contrast, in the Japanese littleneck, Yesso scallop, and common orient clam, cholesterol was just 17.1%, 18.3%, and 18.9% of total sterols, respectively, making them the richest source of non-cholesterol sterols (NCS). Lipids extracted from shellfish species showed ABTS^+•^- and DPPH^•^-scavenging activities. In the cytotoxicity analysis, lipids extracted from the Argentine red shrimp showed the highest cytotoxicity against glioblastoma multiforme T98G cells, with an IC_50_ value of 12.3 µg/mL. The composition and cytotoxicity data reported herein may help explore the nutritional and anticancer potentials of shellfish species.

## 1. Introduction

Shellfish (mollusks and crustaceans) species are the key dietary source of omega-3 (n-3) very-long-chain (VLC)-polyunsaturated fatty acids (PUFAs), mainly eicosapentaenoic (EPA; C20:5n3), and docosahexaenoic acids (DHA; C22:6n3), which play a crucial role in reducing the risk of cancer and cardiovascular diseases (CVD). Moreover, DHA-derived specialized pro-resolving mediators (SPMs, e.g., protectins, resolvins, and maresins) are critically crucial for neonatal brain development and mental and cognitive development [1] and neuroprotection [2,3].

Though with the richness of VLC-n-3 PUFAs, shellfish are considered a vital component of a healthy diet, the high cholesterol content of some species is generally cited as a reason to limit their intake. However, in addition to cholesterol, shellfish species contain a significant amount of other sterols (called non-cholesterols sterols; NCS); some are unique to marine species [4], derived from the food they consume (e.g., microalgae) and from endogenous metabolism. Plant-derived sterols (called phytosterols) are well known to reduce low-density plasma lipoprotein-cholesterol (LDL-C) levels [5,6,7] and thereby lower the risk of CVD. Moreover, as antioxidants, phytosterols are well-known to scavenge harmful reactive oxygen species (ROS) [8]. Furthermore, animal and human studies have demonstrated the anticancer [9] and anti-inflammatory [10] effects of phytosterols. Considering these facts, consuming a phytosterol-rich diet may provide health benefits.

Tocopherols and tocotrienols (collectively known as tocols, tocochromanols, or vitamin E) are key components of plant- and animal-derived lipids, which scavenge ROS, thus protecting lipids from oxidative degradation. Given their functional role in controlling cellular oxidative stress, a diet rich in tocols minimizes the incidence of cancer, CVD, and neurodegenerative diseases [11,12,13].

To the best of our knowledge, only a few detailed studies are available on the sterol composition of shellfish species [4], while most studies reported only cholesterol and few other sterols, such as sitosterol, campesterol, or stigmasterol [14,15,16], while many shellfish species contain still other sterols at significant levels. Moreover, culturing conditions and dietary factors can affect the sterol content of shellfish species harvested from different natural geographical locations [4]. Similarly, the detailed composition of many shellfish species commonly consumed in Korea is not available. Furthermore, similarly to sterols, fatty acids’ composition in shellfish may vary with season [17], sex (e.g., male vs. female crabs) [18], culturing conditions (e.g., wild vs. cultured, and diet quality) [19,20], and several other factors [21].

Considering the above, the present investigation aims to analyze the composition and contents of the major lipophilic compounds, including sterols, fatty acids, and tocols, of shellfish species, commonly consumed in Korea. Sterols were analyzed by gas chromatography (GC)-mass spectrometry (MS), fatty acids by GC-flame ionization detection (FID) and GC-MS, and tocols by high-performance liquid chromatography (HPLC)-diode-array detection (DAD). Moreover, to explore the antitumoral activities of the lipophilic constituents of shellfish species, the cytotoxicity potentials of the extracted lipids were determined against five different human cancer cells, including colon carcinoma (HCT116), epithelial melanoma (A2058), glioblastoma multiforme (T98G), lung carcinoma (A549), and adenocarcinoma (HeLa). The fatty acids, sterols, and tocols composition, and the cytotoxicity data reported herein may help explore shellfish species’ nutritional and anticancer potentials.

## 2. Materials and Methods

### 2.1. Reagents, Standards, and Raw Material

Authentic standards of sterols including cholesterol (>99% purity), cholestanol (>99% purity), brassicasterol (>98% purity), ergosterol (quality level of MQ300), and campesterol (90% purity) and fatty acids (37-Component FAME Mix, CRM47885, quality level of MQ100) were obtained from Merck Ltd., Seoul, South Korea. A mixed-tocols solution containing δ-, γ-, β-, and α-tocotrienol and δ-, γ-, β-, and α-tocopherol (purity adjusted) was purchased from ChromaDex, Inc. (Irvine, CA, USA). All organic solvents used for the extractions were HPLC grade and obtained from Daejung Chemicals and Metals Co., Ltd., Siheung-si, Korea.

A total of eleven shellfish species consumed in Korea were procured from the Garak market, Songpa-gu, Seoul, in October 2019 (Table 1). Two kilograms of each species were brought to the lab, the edible flesh was manually separated and homogenized using a food processor, and 30-g portion was precisely aliquoted to falcon tubes and stored at −80 °C until analysis.

### 2.2. Extraction of Crude Lipids (Lipophilic Compounds)

Crude lipids, containing lipophilic compounds, were extracted from the edible portions of the studied shellfish species following our optimized protocol [22], with minor modifications; it was initially based on a previous report [23]. The detailed extraction procedure is illustrated in Appendix A. The butylated hydroxytoluene (BHT: *w*/*v*; synthetic antioxidant) was added to the extraction solvent to minimize the degradation of lipophilic compounds [24]. The crude lipids utilized for the cell culture analysis were extracted without using the BHT. The extracted crude lipids were utilized to analyze their fatty acids, tocols, and sterols, and to determine their anticancer potentials, as shown in Appendix A. Tocols were analyzed by HPLC without hydrolysis, as it can degrade the tocols [23]. The crude lipids were converted to fatty acid methyl esters (FAMEs) using a commercially available boron trifluoride-methanol solution (14% in methanol; Merck Ltd., Seoul, South Korea) as per the manufacturer’s guidelines, with minor modifications (Appendix B). Similarly, before the GC-MS analysis, the crude lipids were hydrolyzed for sterol analysis, as mentioned in Appendix B [23].

### 2.3. HPLC Analysis of Tocols

The chromatographic separation of tocols was achieved utilizing an HPLC system (Model 1100; Agilent Technologies Canada, Inc., Mississauga, ON, Canada) equipped with a YMC C_30_ column (250 × 4.6 mm, 5 μm; YMC, Wilmington, NC, USA) and a DAD. The solvent system, gradient elution pattern, flow rate, and detection wavelengths were used as previously optimized [25].

### 2.4. FAMEs Determinations by Gas Chromatography (GC)-Flame Ionization Detection (FID) and GC-Mass Spectrometry (MS)

FAMEs were quantitatively analyzed by GC (Agilent 7890B, Agilent Technologies, Santa Clara, CA, USA) equipped with an FID and an SP-2560 capillary column (100 m, 0.20 μm film thickness, 0.25 mm ID; Merck KGaA, Darmstadt, Germany). The thermal program and other FID parameters were used as optimized in recent investigations [26]. For precise identification, compounds’ mass spectra were recorded through the GS-MS system (QP2010 SE; Shimadzu, Japan), following a thermal program of GC-FID analysis. The identities of FAMEs were confirmed by comparing their fragmentation pattern with the authentic standards.

### 2.5. Calculation of Fat Quality Indices

Fat-quality indices, including total polyunsaturated fatty acids (PUFAs)/total saturated fatty acids (SFAs), n-3 PUFAs/n-6 PUFAs, hypocholesterolemic (h)/hypercholesterolemic (H) fatty acids ratios, atherogenic index (AI), and thrombogenic index (TI), were calculated from the fatty acid-profiling results [27,28,29,30]. The h/H, AI, and TI were calculated as per the following equations:

$$h/H=\frac{C18:1+\sum \mathrm{PUFA}}{C14:0+C16:0}$$

$$\mathrm{AI}=\frac{\left(4\;\times \;C14:0\right)+C16:0}{\sum \mathrm{MUFAs}+\sum \mathrm{PUFAs}}$$

$$\mathrm{TI}=\frac{C14:0+C16:0+C18:0}{\left(0.5\;\times \;\sum \mathrm{MUFAs}\right)+\left(0.5\;\times \;\sum n-6\;\mathrm{PUFAs}\right)+\left(3\;\times \;\sum n-3\;\mathrm{PUFAs}\right)+\left(\frac{\sum n-3\;\mathrm{PUFAs}}{\sum n-6\;\mathrm{PUFAs}}\right)}$$

### 2.6. GC-MS Analysis of Sterols

Sterols were analyzed after silylation, utilizing a QP2010 SE GC-MS equipped with a fused silica Rxi-5ms column (30 m, 0.5-μm film thickness, 0.25-mm ID; Restek Corporation, Bellefonte, PA, USA). Helium was used as a carrier gas, maintained at the pressure control flow of 33.5 cm/min (7.8-mL/min total flow). The injector and MS ion source were precisely maintained at 260, while the MS interface was maintained at 280 °C. The column oven temperature was kept at 120 °C for 1 min, then progressively increased to 300 °C with a linear increase of 15 °C/min, and held at 300 °C for 27 min [31]. One µL of samples and standards were injected in a 1:5 split ratio. Their fragmentation pattern was compared with authentic standards and reference databases (NIST08, NIST08S, and Wiley9).

### 2.7. Total Equivalent Antioxidant Capacities (TEAC)

The total equivalent antioxidant capacities (TEAC) of the lipids extracted from the shellfish species was determined using 1,1-diphenyl-2-picrylhydrazyl- (DPPH^•^) and 2,2′-azino-bis(3-ethylbenzothiazoline-6-sulphonic acid)- (ABTS^•+^) scavenging assays following our previously optimized procedures [32], originally based on the studies of Thaipong et al. [33] with minor modifications.

Briefly, for the ABTS^+•^ decolorization assay, 1950 µL of freshly prepared ABTS^+•^ solution was allowed to react with 50 µL (1.5 mg) of extracted lipids for 2 h in the dark. Later, the absorbance was measured at 734 nm, using a spectrophotometer (UV-2550, Shimadzu, Japan). A linear standard calibration curve was prepared using Trolox as standard in the range of 0.01–10 µg/mL. The results are expressed as the mg-of-Trolox equivalents (TE)/g of the lipids.

For the DPPH^•^-scavenging activities, 1950 µL of freshly prepared DPPH^•^ solution (0.1 mM) was allowed to react with 50 µL (1.5 mg) of extracted lipids for 80 min in the dark. Later, the absorbance was measured at 517 nm using the spectrophotometer. A linear standard calibration curve was prepared using Trolox as standard in the range of 0.01–10 µg/mL. The results are expressed as the mg-of-Trolox equivalents (TE)/g of the lipids.

### 2.8. Cytotoxicity Studies

The HCT116 (Colorectal carcinoma), A2058 (Melanoma), A549 (lung carcinoma), T98G (Glioblastoma multiforme), and Hela (Adenocarcinoma) cell lines were purchased from the American Type Culture Collection (ATCC, Manassas, VI, USA). Cells were cultured in McCoy’s 5A Medium, Dulbecco’s Modified Eagle’s Medium, or Minimum Essential Medium Eagle, supplemented with 10% FBS, 1 mM sodium pyruvate, and 100 units/mL penicillin/streptomycin, accordingly, and incubated in a 5%-CO_2_ incubator at 37 °C. Prior to treatment, cells were seeded at 3 × 10^3^ cells/per well density in 96-well plate and incubated overnight. Treatments were performed by carefully washing the cells with Dubelcco’s Phosphate Buffered Saline twice and adding lipids in media without FBS to the washed wells. Lipids were dissolved at a concentration of 20 mg/mL in DMSO, and cells were treated at concentrations ranging from 10 to 100 µg/mL in 0.5% DMSO for 24 h. Cytotoxicity was assayed by adding 10 µL of WST-1 EZ-cytox (DoGen, Suwon, Korea) per well with 90 µL of media without FBS and incubated at 37 °C for 40 min. The absorbance at 450 nm and reference wavelength at 600 nm were measured using a Microplate Spectrophotometer (Biotek, VT, USA). Experiments were performed in three technical repeats for more than three biological repeats. The most representative biological repeats were used to calculated IC_50_ concentration by mean of the AAT-Bioquest^®^ online tool.

### 2.9. Statistical Analysis and Quality Control

A total of three replicate extraction and analysis were performed for each shellfish species. The results were analyzed using IBM SPSS statistics (version 25; IBM Corp., Armonk, NY, USA) employing a one-way analysis of variance (ANOVA), considering a significance level of 0.05 (Turkey HSD).

The use of the GC-MS method for quantifying sterols was recently validated in terms of accuracy, linearity, precision, and stability [26]. The recovery of sterols was precisely monitored and normalized using 5β-cholestan-3α-ol as an internal standard.

## 3. Results and Discussion

### 3.1. Qualitative Confirmation of Sterols by GC-MS

**Figure 1:**
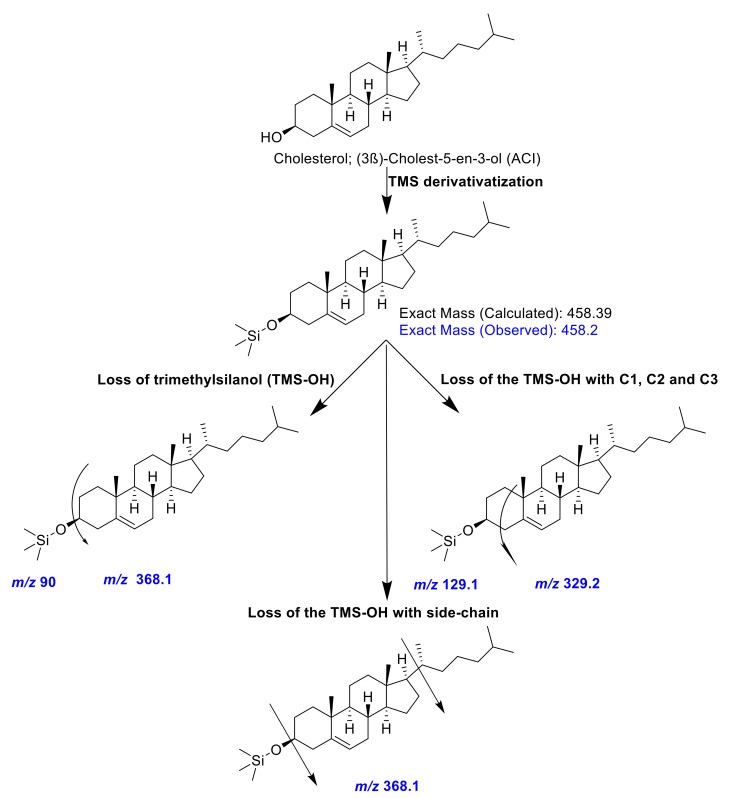
A representative mass-fragmentation pattern of cholesterol (trimethylsiloxy [TMS] derivative) observed in the present investigation. The colored text explains the observed *m/z* values.

**Figure 2:**
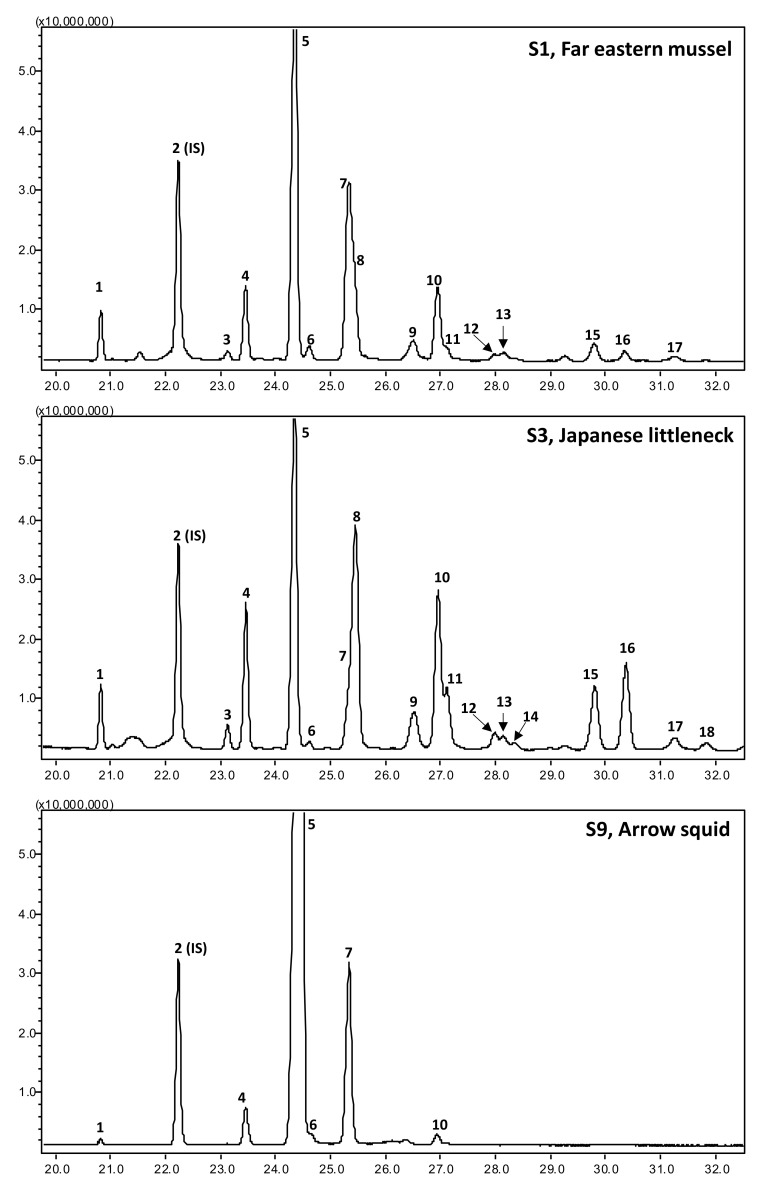
The representative gas chromatography (GC), mass spectrometry (MS), and total ion chromatograms (TIC) of the sterols of the far eastern mussel, Japanese littleneck, and arrow squid. The peak numbers (1 to 18) correspond to Table 2. IS: internal standard.

In this study, 11 shellfish species were analyzed for their compositions of sterols utilizing GC-MS after derivatization with trimethylsiloxy groups [TMS; −O-Si(CH_3_)_3_]. In the mass spectrometric identification of sterols, the loss of trimethylsilanol (TMS-OH) is a common feature, resulting in dominant ion fragments at *m/z* [M-90]^•+^ [34]. The Δ5-steryl (TMS derivatives) give a characteristic fragmentation involving the loss of the C-l, C-2, and C-3 the sterol. The A-ring with the TMS-group yielded intense ions at *m*/*z* 129 for the TMS derivative-containing fragment and *m*/*z* [M-129]^•+^ for the remaining portion of the sterol compound (Figure 1 and Figure 2) [34].

In the present study, a total of 18 sterol compounds were detected among the studied shellfish species (Table 2). Among these sterols, 14 compounds were identified with the help of authentic standards, and NIST08, NIST08S, WILAY8, and WILAY 09 mass spectra library. Moreover, the mass spectral and gas chromatographic patterns reported in previous studies were found valuable in identifying the sterols [4,35]. Especially, stigmasterol and poriferasterol have nearly similar retention time and mass spectrum profiles, as do sitosterol and clionasterol [4], making it difficult to confirm the identity by GC-MS. Hence, peak assignments for these sterols were based on the previous reports of the isomers occurring in shellfish species [4,35].

**Figure 3:**
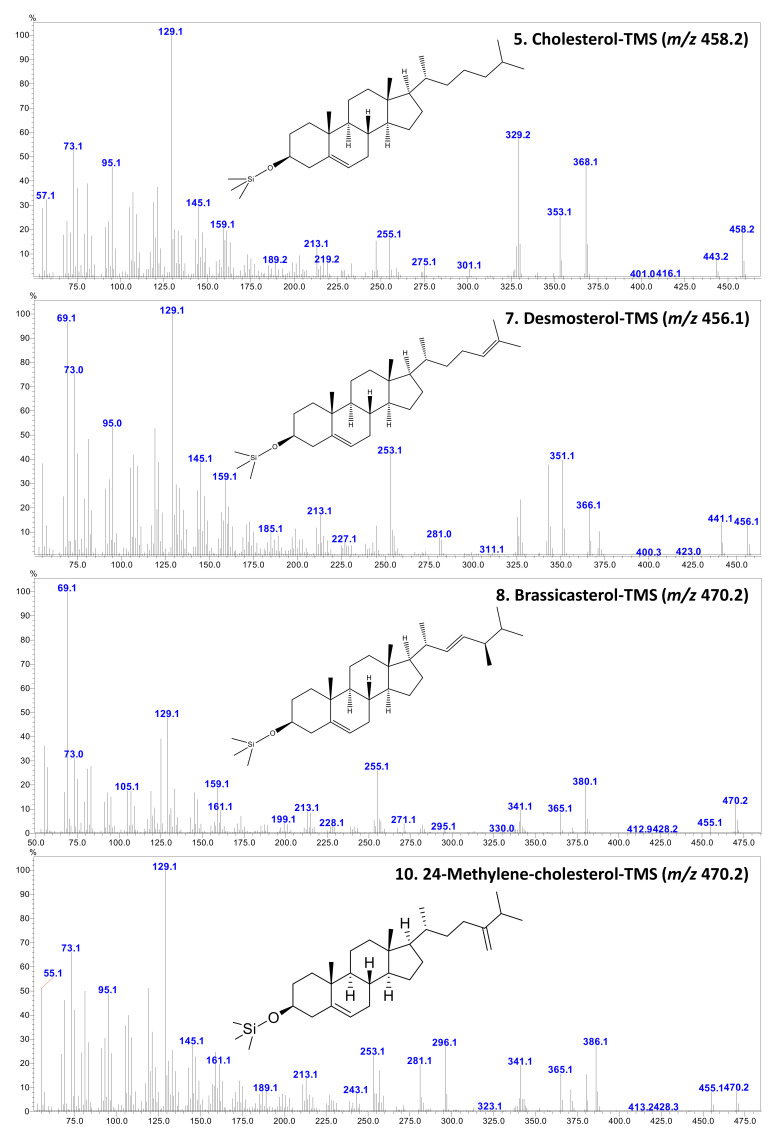
A representative mass spectrometric fragmentation pattern of major sterols (trimethylsiloxy [TMS] derivatives) identified and quantified from the shellfish species. The numbers 5, 7, 10, and 10 correspond to peak numbers in Table 2 and Figure 1.

The representative total ion chromatograms (TIC) and mass spectrum of major sterols identified in this study are given in Figure 2 and Figure 3, respectively. In addition, the mass spectrum of four minor unidentified sterols are given in Appendix C. In the present study, mass spectrum and elution order were validated for all identifications; however, the possibilities of sterol compound co-elution and isomeric variation suggest that the presented sterol identities should be considered tentative. Further analysis with the support of NMR could provide further information for confirming their identities.

### 3.2. Sterol Contents in Studied Mollusk and Crustacea

In the present study, the studied shellfish species showed significant variation in their sterol compositions and contents. Cholesterol was the most dominant sterol in most of the studied shellfish species (Table 3). The highest cholesterol content was recorded in arrow squid (154.4 mg/100 g) and Argentine red shrimp (110.3 mg/100 g), which accounted for 92.6% and 90.3% of total sterols, respectively. In contrast, in the Japanese littleneck, Yesso scallop, and common orient clam, cholesterol was just 17.1%, 18.3%, and 18.9% of total sterols, respectively, which makes them the richest source of NCS. The brassicasterol, 24-methylene-cholesterol, 22-dehydrocholesterol, and isofucosterol were the dominant NCS among these species. In addition, a significant amount of desmosterol was recorded in the far eastern mussel (19.4 mg/100 g), arrow squid (16.8 mg/100 g), and horned turban (14.7 mg/100 g).

Only a few detailed studies are available on the sterol compositions of shellfish species [4], while most studies reported cholesterol and few other sterols [14,15,16]. The cholesterol contents recorded in the present investigation are in agreement with previous studies on the Japanese littleneck and Yesso scallop [14]. Philips et al. [4] recorded the sterol composition of shellfish species for the United States Department of Agriculture (USDA) National Nutrient Database. In this USDA study, the high content of cholesterol (129 mg/100 g FW) was recorded in shrimp, whose total sterol content was 134 mg/100 g FW. In agreement therewith, we also recorded a significant amount of cholesterol (110.3 mg/100 g FW) in the Argentine red shrimp, whose total sterol content was 122.2 mg/100 g FW. Similarly, Bragagnolo and Rodriguez-Amaya [16] recorded a substantial amount of cholesterol (114–134 mg/100 g) in several species of wild shrimp.

The high contents of cholesterol is often cited as a reason to limit the consumption of shellfish species, as cholesterol is generally considered a risk factor for developing CVD [36], and known to initiate pathophysiological angiogenesis [37]. The American Heart Association (AHA) dietary guidelines (2000 edition) advised the consumption of <300 mg/day of cholesterol to minimize elevations in blood cholesterol [38]. Interestingly, in the 2015 edition of dietary guidelines, these restrictions were not included [39]. Interestingly, even if the recommendation of the consumption of <300 mg/day of cholesterol is considered, a serving size of 85 g of far eastern mussel, Japanese little neck, Yesso scallop, common orient clam, or pacific oyster would contribute ≈10% of the daily maximum, while the remaining species would contribute ≈19% (horned turban, Gazmi crab, and long arm octopus), 31% (Argentine red shrimp), and 43% (arrow squid) of daily value.

### 3.3. Fatty Acids

**Figure 4:**
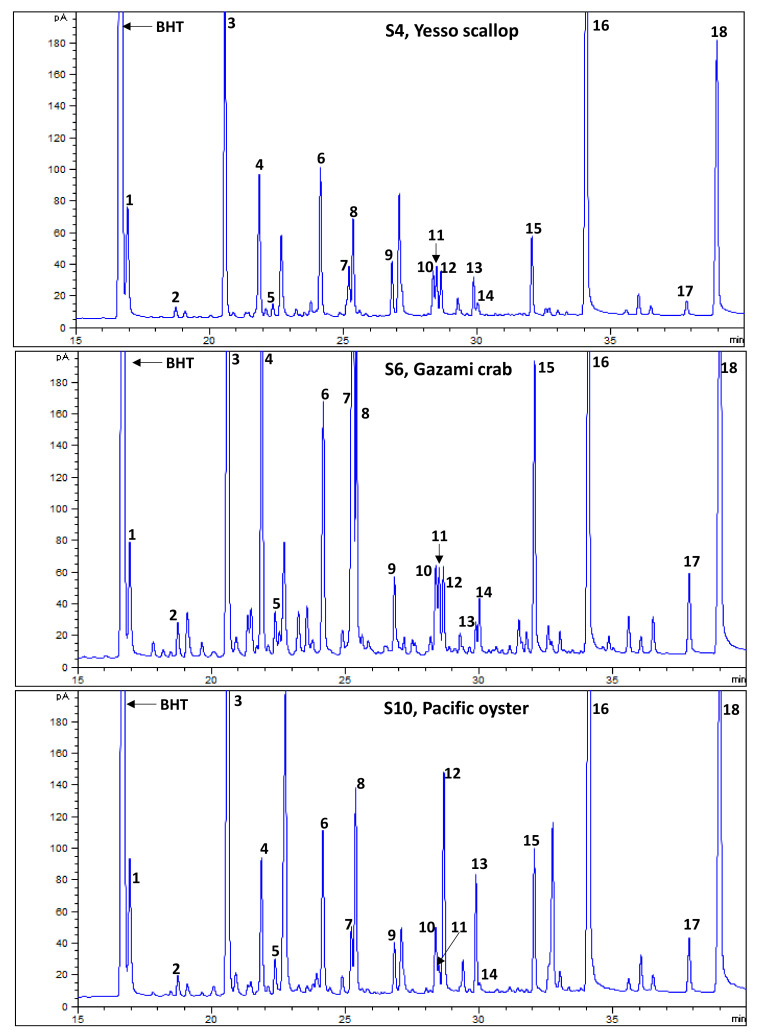
The representative gas chromatography (GC) flame ionization detection (FID) chromatograms of fatty acids and methyl esters (FAMEs) of the Yesso scallop, Gazami crab, and oyster. The peak numbers (1 to 18) correspond to Table 4.

In the present investigation, 18 fatty acids were identified from the edible flesh of shellfish species (Table 4, Figure 4). Among the studied species, DHA, EPA, and palmitic acid (C16:0) were most dominant. Together, these three fatty acids accounted for 30.7% (abalone) to 81.7% (arrow squid) of the total fatty acids. Amid the highest proportions of palmitic acid (29.3%), the total SFAs were recorded highest (37.6) in the lipids extracted from arrow squid. Interestingly, the highest amount of DHA (39.6%) was also recorded from arrow squid.

With the presence of the highest amount of α-linolenic (C18:3n3; 5.85%), stearidonic (C18:4n3; 2.60%), and a fairly good amount of EPA (24.9%), and DHA (17.9%), the highest amount of total PUFAs (58.1% of total fatty acids) were recorded from the Pacific oyster. The representative GC-FID chromatograms of arrow squid and pacific oyster are given in Figure 4.

The lipid composition recorded in the present investigation are in agreement with previous studies on the far eastern mussel [40], Japanese littleneck [14], Yesso scallop [14], abalone [17], and Argentine red shrimp [22]. However, contrasting results were obtained for some shellfish species. For instance, Saito and Aono [19] recorded only a trace amount of DHA in the different lipid fractions of wild and cultured horned turban viscera. However, in the present investigation, we recorded a significant amount of DHA (11.5%) in the horned turban. Similarly, Lu et al. [18] recorded a significantly higher amount of EPA and DHA in the edible viscera of female Gazami crab (7.72% EPA and 16.5% DHA), compared with the male Gazami crab (3.47% EPA and 7.30% DHA). We recorded 13.4% EPA and 17.1% DHA in the male Gazami crab (female Gazami crab not investigated) in the present investigation.

Fatty acid compositions in seafood may vary with season [17], sex (e.g., male vs. female crab) [18], culturing conditions (e.g., wild vs. cultured, diet quality) [19,20], and several other factors [21].

Shellfish species are well known to contain a significant amount of EPA, DHA, and other health-beneficial n-3 fatty acids [41,42,43]. In the present study, all the studied shellfish showed the presence of a significant amount of DHA, except abalone. Previous studies have also shown the complete absence of DHA in abalone species [17]. In the present study, abalone showed the unusual presence of the highest amount of docosapentaenoic acid (C22:5n3); a most abundant n-3 VLC-PUFA in the human brain after DHA, beneficial for elderly neuroprotection and early-life development [44].

### 3.4. Fatty Acid Indices

The consumption of PUFAs, especially n-3 PUFAs in appropriate proportions, is highly beneficial in reducing risk to CVD and many other chronic diseases [45,46]. Among the studied species, the highest amount of total n-3 PUFAs were recorded from arrow squid (53.2%) and pacific oyster (53.0%) (Table 5). However, with the presence of the highest amount of palmitic acid (29.3% of total lipids), the highest total SFAs (37.6%) were also recorded in arrow squid. In view of the risk of CVD and other chronic diseases associated with the consumption of SFAs [46], fats with PUFAs/SFAs ratios of greater than 0.45 are considered safe for human consumption [28]. In the present study, the PUFAs/SFAs ratios ranged from 1.25 (abalone) to 2.18 (Yesso scallop) (Table 5), showing that lipids from all the studied shellfish species are healthy for consumption. Furthermore, the fats with higher ratios of h/H fatty acids and lower TI and AI, are suggested for minimizing the risk of CVD [27]. In the present study, a significant difference was recorded for h/H fatty acid ratios, TI and AI values of fat obtained from the shellfish species. The significantly highest h/H fatty acid ratios of 4.41 and 4.36 were recorded from Gazami crab and Yesso scallop, respectively. Similarly, the lowest TI (0.15) and AI (0.33) were also recorded also recoded from the Yesso scallop, and Gazmi crab, respectively. These observations indicate the lipids obtained from the Yesso scallop and Gazmi crab are least hypercholesteromic, atherogenic and thrombogenic, hypercholestromic.

### 3.5. Tocols Composition

**Figure 5:**
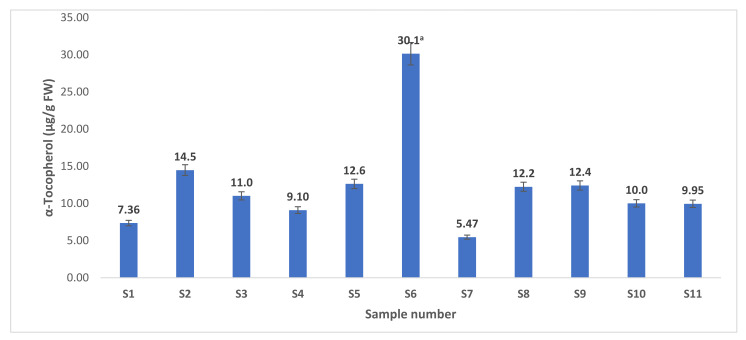
The contents of α-tocopherol (µg/g fresh weight) of the studied shellfish species. Values are mean ± standard deviation of three replicates. ^a^ The mean value is significantly (*p* < 0. 05, Turkey HSD) highest among the studied species. The sample numbers (S1 to S11) correspond to Table 1.

This study screened lipids obtained from the shellfish species for tocols composition by HPLC-DAD. A significant amount of α-tocopherol was recorded in all studied samples, with the highest content in the Gazami crab (30.1 µg/g FW) (Figure 5), while other forms were not detected in a substantial amount.

Only a few studies are available on the tocopherol contents of shellfish species. In raw clams (unknown species), Kuhnlein et al. [11] recorded 5.7 µg/g of α-tocopherol, However, in the present study, we recorded 12.6 µg/g of α-tocopherol in the common orient clam. Tocopherols are potent free radical scavengers that play a crucial role in minimizing oxidative stress-related diseases, including cancer, cardiovascular, and neurodegenerative diseases [11,12]. Thus, a diet rich in tocopherols may provide protection against these chronic diseases.

### 3.6. Antioxidant Activity of Lipids Extracted from Shellfish Species

**Figure 6:**
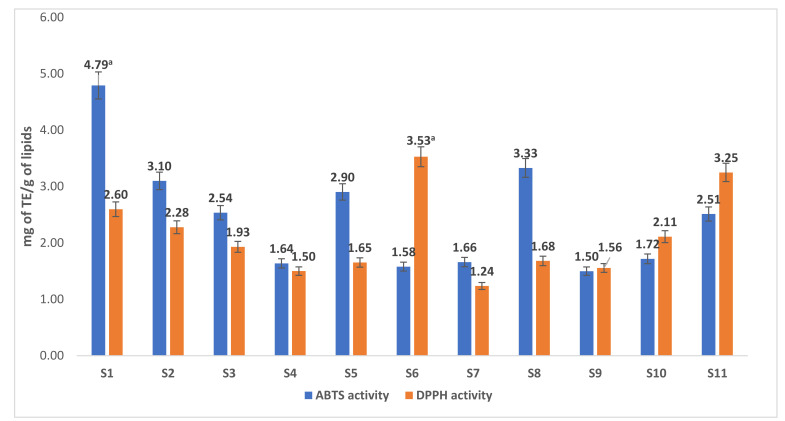
The antioxidant activity (mg of Trolox equivalent (TE)/g of lipids) of studied shellfish species. Values are mean ± standard deviation of three replicates. ^a^ The mean value is significantly (*p* < 0. 05, Turkey HSD) highest among the studied species. The sample numbers (S1 to S11) are correspondent to Table 1.

Results of DPPH^+•^- and ABTS^+•^-scavenging activities of lipids extracted from the shellfish species are shown in Figure 6. Among the studied shellfish species, the highest ABTS^+•^-scavenging activities of 4.79 mg TE/g of lipids were recorded from the far eastern mussel. In contrast, the highest DPPH^+•^-scavenging activities of 3.53 mg TE/g of lipids were recorded from the Gazami crab. Surprisingly, in this study, the highest content α-tocopherol was also recorded in the Gazami crab.

Although both DPPH and ABTS assays are commonly classified as electron transfer (ET) reactions, these two radicals actually may be deactivated either by direct reduction through ET mechanisms or by radical quenching via hydrogen atom transfer (HAT) [47]. The higher DPPH free radical scavenging activities of the Gazmi crab is probably due to the higher contents of α-tocopherol, which is considered a potent free radical scavenger [12]. However, the higher ABTS activities of far eastern mussel (containing a low amount of α-tocopherol), suggesting that other lipophilic compounds are probably responsible for these activities. Overall, the strong free radical scavenging activities of shellfish lipids may help in minimizing oxidative stress-related diseases [11,12].

### 3.7. Anticancer Potential of Lipids Extracted from Shellfish Species

The few reports on the effect of shellfish lipids in cancer are controversial. While n-3 PUFAs, the most well-known fatty acids in shellfish lipids, was previously reported to have antitumoral effects [43,44], it was also reported that shellfish intake increased the risk of head and neck cancer [45].

The lipophilic compounds recorded in the present investigation, such as α-tocopherol [48], n-3 PUFAs (especially EPA and DHA) [49], and sterols [9], have shown anticancer effects. In the present investigation, the brassicasterol, 24-methylene-cholesterol, 22-dehydrocholesterol, and isofucosterol were recorded as the dominant NCSs. Brassicasterol-rich lipids from edible *Hippocampus abdominalis* have shown cytotoxicity against LNCaP human prostate cancer cells [50]. Lipid fractions rich in 22-dehydrocholesterol, brassicasterol and other marine animal specific sterols from the Persian Gulf sponge, *Axinella sinoxea,* have also shown cytotoxicity against human lymphoblastic leukemia (MOLT-4), human breast adenocarcinoma (MCF-7), and human colorectal adenocarcinoma (HT-29) cells.

**Figure 7:**
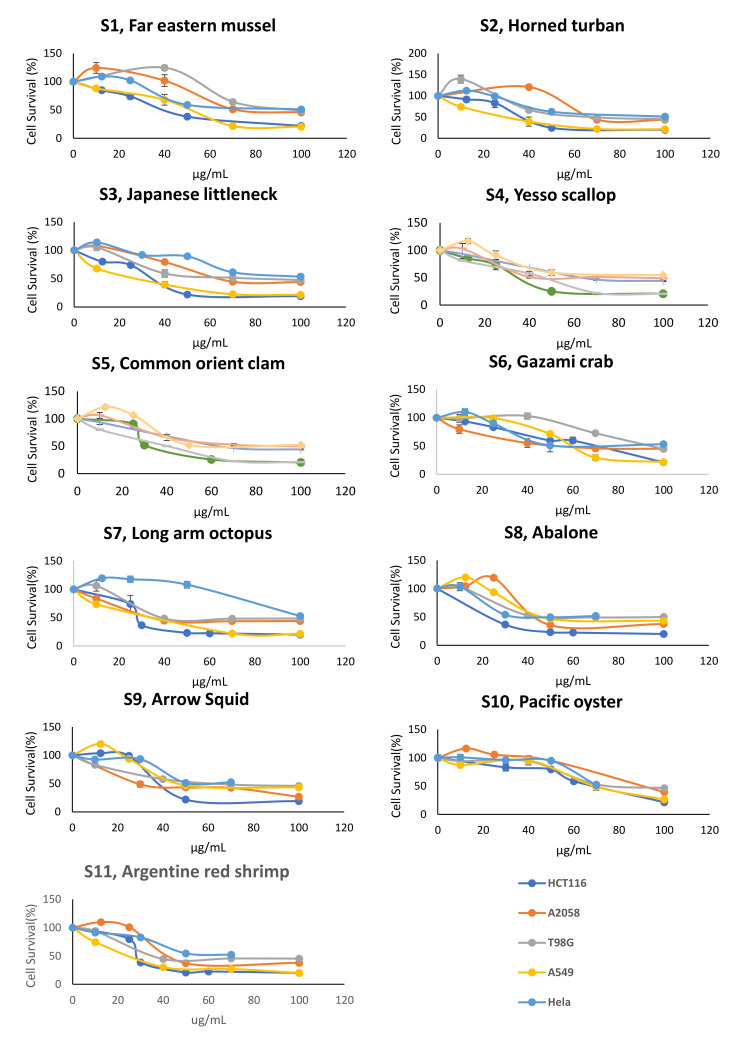
Cell survival analysis of shellfish lipids treatment to cancer cell lines. Values are mean standard ± standard deviation of three replicates for cell survival in % compared to control (0.5% DMSO) treatment for 24 h.

**Figure 8:**
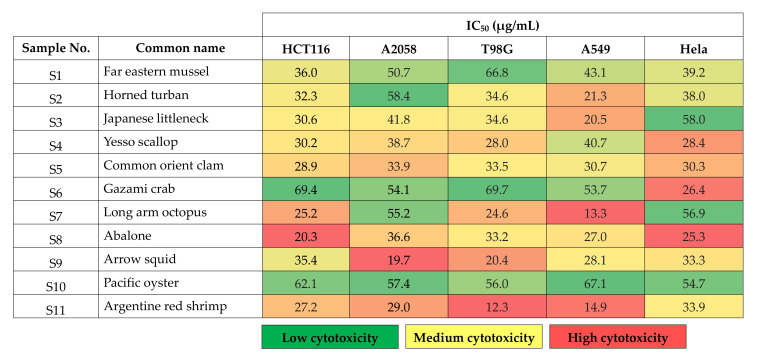
IC50 of eleven shellfish extracted lipids. The green, yellow, and red color explains the low, medium, and high cytotoxicity, respectively.

In our study, we found that most of the lipids assayed have anticancer activities, while, at low concentrations, specific lipids showa biphasic response, that is, a hormesis effect, for specific cell lines (Figure 7), suggesting that such response is specific to concentration threshold as well as to cancer type. Among the eleven types of studied shellfish, the Argentine red shrimp lipid extract was the most cytotoxic lipid across all cancer cells investigated, while the Gazami crab’s lipids were relatively less cytotoxic to cancer cells, except for Hela cells, as can be observed from the IC_50_ obtained (Figure 8).

## 4. Conclusions

The results of the present investigation indicated that the contents and composition of sterols, fatty acids, and tocopherols varied significantly among shellfish species. The highest n-3 PUFAs were recorded from arrow squid and pacific oysters, accounting for 53.2% and 53.0% of total fatty acids, respectively. However, the highest cholesterol content was recorded in arrow squid (154.4 mg/100 g; 92.6% of total sterols). In contrast, in the Japanese littleneck, Yesso scallop, and common orient clam, cholesterol was just 17.1%, 18.3%, and 18.9% of total sterols, respectively, which makes them the richest source of NCSs. The fat-quality indices indicated that lipids obtained from the Yesso scallop and Gazmi crab are least hypercholesteremic, atherogenic, and thrombogenic.

Lipids extracted from shellfish species showed ABTS^+•^-and DPPH^+•^-scavenging activities. The highest ABTS^+•^-scavenging activities were recorded from the far eastern mussel, while the lipids from the Gazami crab showed the highest DPPH^+•^-scavenging activities.

In the cytotoxic studies, lipids extracted from shellfish species showed varied levels of cytotoxicity against the studied cancer cells. The lipids extracted from the Argentine red shrimp showed the highest cytotoxicity against glioblastoma multiforme (T98G) cells, with an IC50 of 12.3 µg/mL. In contrast, lipids of the long arm octopus were the most cytotoxic to lung carcinoma (AS49) cells, with an IC_50_ of 13.3 µg/mL.

The composition of major lipophilic compounds and cytotoxicity data against several cancer types of cancer cells reported herein may be helpful in exploring the nutritional and anticancer potential of shellfish species.

The presence of a wide range of sterols (including isomeric forms) in shellfish species makes the qualitative analysis complicated. In the future, investigation with the aid of NMR could provide further information to confirm the identities. Moreover, sampling from several locations of the country can provide more comprehensive information on the contents of these nutritionally vital lipophilic constituents and their anticancer potential.

## Figures and Tables

**Table 1:** List of shellfish species used in the present investigation.

Sample No.	Common Name	Zoological Name
S1	far eastern mussel	*Mytilus coruscus* (Gould, 1861)
S2	horned turban (topshell)	*Turbo cornutus* (Lightfoot, 1786)
S3	Japanese littleneck	*Venerupis philippinarum* (Adams & Reeve, 1850)
S4	Yesso scallop	*Mizuhopecten yessoensis* (Jay, 1857)
S5	common orient clam	*Meretrix lusoria* (Roeding 1798)
S6	Gazami crab	*Portunus trituberculatus* (Miers, 1876)
S7	long-arm octopus	*Octopus minor* (Sasaki, 1920)
S8	abalone	*Haliotis discus*, Linnaeus, 1758
S9	arrow squid	*Heterololigo bleekeri* (Keferstein, 1866)
S10	Pacific oyster	*Magallana gigas* (Thunberg, 1793)
S11	Argentine red shrimp	*Pleoticus muelleri* (Bate, 1888)

**Table 2:** List of identified and quantified sterols from the studied species.

Peak No.	Sterol	CAS Registry Number ^c^	Another Name	Calculated Exact Mass of Sterol-TMS ^d^	Observed Exact Mass	RT (min)
1	norcholestadienol ^a^	38788-81-7	(22E)-24-Norcholesta-5,22-dien-3ß-ol	442.34	442.1	20.81
3	occelasterol ^a^	54278-89-6	(22Z)-27-Norergosta-5,22-dien-3β-ol	456.38	456.1	23.11
4	22-dehydrocholesterol ^a^	34347-28-9	Cholesta-5,22E-dien-3β-ol	456.38	456.1	23.44
5	cholesterol ^b^	57-88-5	Cholest-5-en-3-ol	458.39	458.2	24.37
6	cholestanol ^b^	80-97-7	(5α)-cholestan-3ß-ol (Dihydrocholesterol)	460.41	460.2	24.60
7	desmosterol ^a^	313-04-2	Cholesta-5,24-dien-3ß-ol	456.38	456.1	25.32
8	brassicasterol ^b^	474-67-9	Ergosta-5,22E-dien-3ß-ol	470.39	470.2	25.41
9	ergosterol ^b^	57-87-4	(22E)-ergosta-5,7,22-trien-3ß-ol	468.38	468.1	26.49
10	24-methylene-cholesterol ^a^	474-63-5	24-Methylene-cholest-5-en-3ß-ol	470.39	470.2	26.92
11	campesterol ^b^	474-62-4	Campest-5-en-3ß-ol (24α-methyl-cholesterol)	472.41	472.2	27.07
12	poriferasterol ^a^	481-16-3	(3β,22E,24R)-Stigmasta-5,22-dien-3-ol	484.41	484.2	27.96
13	unidentified 1	-	-	-	468.1	28.12
14	unidentified 2	-	-	-	470.1	28.32
15	clionasterol ^a^	83-47-6	(24S)-Stigmast-5-en-3β-ol (γ-Sitosterol)	486.43	486.2	29.77
16	isofucosterol ^a^	481-14-1	(24Z)-Ethylidene-cholest-5-en-3b-ol	484.04	484.1	30.33
17	unidentified 3	-	-	-	484.1	31.22
18	unidentified 4	-	-	-	482.1	31.81

^a^ Identified with the help of mass spectra libraries (NIST08, NIST08S, WILAY8, and WILAY 09) and mass spectra reported in previous studies; these sterols were quantified with the standard curve of structurally related sterols. ^b^ Identified and quantified with the help of authentic standards. ^c^ Source: https://scifinder-n.cas.org/ (accessed on 15 October 2021) ^d^ Calculated using ChemBioDraw Ultra version 14.0.0.117 (Perkin Elmer Inc., Waltham, MA, USA). TMS: derivatization with trimethylsiloxy groups [TMS; −O-Si(CH_3_)_3_]. RT: retention time.

**Table 3:** Sterol composition of shellfish species.

Peak No.	Sterol	S1	S2	S3	S4	S5	S6	S7	S8	S9	S10	S11
1	norcholestadienol	3.30 ± 0.26	2.85 ± 0.18	4.03 ± 0.23	5.17 ± 0.26 ^a^	4.29 ± 0.15	0.68 ± 0.02	0.54 ± 0.02	n.d.	0.42 ± 0.04	5.08 ± 0.09 ^a^	0.39 ± 0.04
3	occelasterol	0.68 ± 0.02	0.94 ± 0.07	1.88 ± 0.09	2.54 ± 0.11 ^a^	1.07 ± 0.03	n.d.	n.d.	n.d.	n.d.	1.64 ± 0.03	n.d.
4	22-dehydrocholesterol	6.23 ± 0.74	7.15 ± 0.55	11.3 ± 0.67 ^a^	8.93 ± 0.47	7.77 ± 0.34	3.48 ± 0.11	3.05 ± 0.04	0.30 ± 0.01	3.34 ± 0.10	9.9 ± 0.28	1.76 ± 0.02
5	cholesterol	38.12 ± 4.60	67.8 ± 7.89	32.9 ± 2.57	32.2 ± 2.12	33.2 ± 1.90	65.6 ± 3.32	71.6 ± 3.07	107.0 ± 4.51	154.4 ± 7.42 ^a^	34.1 ± 0.95	110.3 ± 3.70
6	cholestanol	1.18 ± 0.12	0.50 ± 0.00	0.64 ± 0.02	1.52 ± 0.08	n.d.	n.d.	0.62 ± 0.10	1.03 ± 0.01	0.68 ± 0.03	2.53 ± 0.06 ^a^	1.17 ± 0.06
7	desmosterol	19.4 ± 1.78 ^a^	14.7 ± 1.60	6.11 ± 0.56	1.93 ± 0.31	1.32 ± 0.25	1.54 ± 0.02	2.72 ± 0.02	3.87 ± 0.25	16.8 ± 0.29	6.22 ± 0.16	1.07 ± 0.06
8	brassicasterol	5.04 ± 0.61	6.88 ± 0.70	22.6 ± 1.23 ^a^	11.5 ± 0.65	12.8 ± 0.29	n.d.	n.d.	n.d.	n.d.	15.9 ± 3.38	n.d.
9	ergosterol	2.92 ± 0.15	3.43 ± 0.42	5.26 ± 0.38 ^a^	0.74 ± 0.14	1.25 ± 0.09	n.d.	n.d.	n.d.	n.d.	n.d.	n.d.
10	24-methylene-cholesterol	7.63 ± 0.56	6.61 ± 0.55	17.2 ± 1.26	22.7 ± 0.72 ^a^	19.2 ± 1.18	2.22 ± 0.11	1.16 ± 0.02	1.72 ± 0.02	1.04 ± 0.02	17.9 ± 0.66	1.61 ± 0.34
11	campesterol	0.87 ± 0.01	6.25 ± 0.41 ^a^	4.85 ± 0.31	2.19 ± 0.41	6.35 ± 0.10 ^a^	1.23 ± 0.07	0.57 ± 0.03	0.78 ± 0.17	n.d.	2.53 ± 0.74	0.00
12	poriferasterol	0.58 ± 0.06	0.60 ± 0.01	1.63 ± 0.11	0.63 ± 0.03	1.89 ± 0.07 ^a^	n.d.	n.d.	n.d.	n.d.	0.95 ± 0.07	n.d.
13	unidentified 1	0.67 ± 0.03	n.d.	0.67 ± 0.01	0.61 ± 0.06	n.d.	n.d.	n.d.	n.d.	n.d.	1.45 ± 0.07 ^a^	n.d.
14	unidentified 2	n.d.	2.12 ± 0.36 ^a^	0.88 ± 0.06	n.d.	n.d.	n.d.	n.d.	n.d.	n.d.	0.00	n.d.
15	clionasterol	2.34 ± 0.14	4.59 ± 0.30	8.48 ± 0.33 ^a^	3.84 ± 0.18	7.64 ± 0.11	0.59 ± 0.01	0.00	0.82 ± 0.04	n.d.	5.88 ± 0.17	n.d.
16	isofucosterol	1.17 ± 0.01	2.56 ± 0.17	10.6 ± 0.56	4.14 ± 0.19	12.7 ± 0.43 ^a^	0.22 ± 0.05	n.d.	n.d.	n.d.	5.65 ± 0.15	n.d.
17	unidentified 3	0.83 ± 0.01	1.93 ± 0.30 ^a^	1.85 ± 0.15 ^a^	n.d.	1.39 ± 0.11	n.d.	n.d.	n.d.	n.d.	n.d.	n.d.
18	unidentified 4	n.d.	0.42 ± 0.05	1.01 ± 0.06 ^a^	n.d.	0.87 ± 0.05	n.d.	n.d.	n.d.	n.d.	n.d.	n.d.
	total sterols	140.4 ± 12.9	172.2 ± 16.2 ^a^	192.3 ± 12.1 ^a^	176.1 ± 8.26 ^a^	176.2 ± 6.84 ^a^	85.8 ± 3.05	88.3 ± 2.62	115.5 ± 4.93	182.9 ± 8.42 ^a^	185.9 ± 0.70 ^a^	122.2 ± 4.07
	total non-cholesterol sterols	102.3 ± 8.31	104.3 ± 8.31	159.4 ± 9.51 ^a^	144.0 ± 6.15	143.0 ± 4.95	20.2 ± 0.27	16.8 ± 0.45	8.52 ± 0.41	28.6 ± 1.00	151.8 ± 0.25	11.9 ± 0.36
	% non-cholesterol sterols	72.9 ± 0.79	60.7 ± 0.89	82.9 ± 0.26 ^a^	81.7 ± 0.35 ^a^	81.2 ± 0.35 ^a^	23.6 ± 1.15	19.0 ± 1.08	7.37 ± 0.04	15.6 ± 0.17	81.6 ± 0.44 ^a^	9.7 ± 0.03

Values (mg/100 g of fresh weight) are mean ± standard deviation from an average of three determinations. n.d.; ^a^ The mean value is significantly (*p* < 0.05; Turkey HSD) highest among the studied species. The peak numbers correspond to those used in Table 2. The sample numbers (S1 to S11) are correspondent to Table 1.

**Table 4:** Fatty acid composition of shellfish species.

Peak No	FAME	RT	S1	S2	S3	S4	S5	S6	S7	S8	S9	S10	S11
1	C14:0 (myristic)	16.91	5.81 ± 0.34	4.36 ± 0.14	2.02 ± 0.20	5.36 ± 0.00	3.28 ± 0.10	2.59 ± 0.01	1.84 ± 0.01	8.77 ± 0.00 ^a^	2.03 ± 0.00	4.21 ± 0.02	2.35 ± 0.11
2	C15:0 (pentadecanoic)	18.72	0.58 ± 0.01 ^a^	0.75 ± 0.00 ^a^	0.53 ± 0.02 ^a^	0.47 ± 0.00 ^a^	0.50 ± 0.00 ^a^	0.75 ± 0.00 ^a^	1.12 ± 1.00 ^a^	0.76 ± 0.01 ^a^	0.42 ± 0.00 ^a^	0.58 ± 0.00 ^a^	0.69 ± 0.09 ^a^
3	C16:0 (palmitic)	20.56	17.3 ± 0.20	12.5 ± 0.24	16.4 ± 0.04	14.16 ± 0.05	19.8 ± 0.04	14.53 ± 0.05	18.1 ± 0.20	18.9 ± 0.05	29.3 ± 0.09 ^a^	18.2 ± 0.00	17.7 ± 0.27
4	C16:1 (palmitoleic)	21.84	8.73 ± 0.04	5.91 ± 0.05	7.47 ± 0.05	6.16 ± 0.26	10.16 ± 0.04 ^a^	8.45 ± 0.00	1.18 ± 0.10	1.60 ± 0.03	0.45 ± 0.00	3.81 ± 0.12	4.34 ± 0.05
5	C17:0 (heptadecanoic)	22.34	1.00 ± 0.02	0.98 ± 0.06	1.33 ± 0.01	0.51 ± 0.06	1.49 ± 0.01	0.88 ± 0.01	1.63 ± 0.02 ^a^	0.57 ± 0.00	0.67 ± 0.00	1.02 ± 0.04	0.95 ± 0.07
6	C18:0 (stearic)	24.12	5.50 ± 0.05	7.86 ± 0.07	7.65 ± 0.06	5.95 ± 0.00	5.85 ± 0.23	5.68 ± 0.00	8.92 ± 0.11 ^a^	5.79 ± 0.02	5.22 ± 0.01	4.59 ± 0.07	5.05 ± 0.04
7	C18:1n9c (oleic)	25.19	1.09 ± 0.02	1.84 ± 0.05	2.62 ± 0.02	2.20 ± 0.24	2.96 ± 0.02	15.21 ± 0.02	2.41 ± 0.04	5.69 ± 0.04	1.54 ± 0.01	1.71 ± 0.03	16.49 ± 0.20 ^a^
8	C18:1n7c	25.34	3.10 ± 0.01	2.21 ± 0.02	3.45 ± 0.02	3.93 ± 0.01	2.94 ± 0.01	5.49 ± 0.01	2.85 ± 0.02	9.42 ± 0.00 ^a^	1.39 ± 0.00	5.26 ± 0.02	3.71 ± 0.06
9	C18:2n6c (linoleic)	26.81	1.62 ± 0.01	3.19 ± 0.15 ^a^	0.61 ± 0.01	2.07 ± 0.02	0.78 ± 0.01	1.43 ± 0.01	0.40 ± 0.12	2.58 ± 0.02	0.32 ± 0.02	1.30 ± 0.02	1.68 ± 0.15
10	C20:1n9 (cis-11-eicosenoic)	28.33	2.06 ± 0.00	4.36 ± 0.03 ^a^	3.00 ± 0.03	1.76 ± 0.02	2.20 ± 0.05	1.84 ± 0.00	0.50 ± 0.03	3.81 ± 0.00	0.16 ± 0.01	1.83 ± 0.05	0.38 ± 0.10
11	C20:1n7c	28.47	3.25 ± 0.00	2.73 ± 0.01	1.55 ± 0.01	1.94 ± 0.01	1.57 ± 0.05	1.56 ± 0.02	4.85 ± 0.00 ^a^	1.05 ± 0.01	2.74 ± 0.02	0.64 ± 0.05	1.06 ± 0.00
12	C18:3n3 (α-linolenic)	28.63	2.51 ± 0.01	2.76 ± 0.01	2.72 ± 0.03	1.87 ± 0.02	5.05 ± 0.06	1.55 ± 0.00	0.58 ± 0.05	2.37 ± 0.01	0.17 ± 0.01	5.85 ± 0.04 ^a^	1.32 ± 0.01
13	C18:4n3 (stearidonic)	29.85	1.60 ± 0.00	0.99 ± 0.01	1.35 ± 0.00	1.33 ± 0.01	1.74 ± 0.00	0.54 ± 0.00	0.07 ± 0.01	0.85 ± 0.00	0.06 ± 0.00	2.60 ± 0.00 ^a^	0.56 ± 0.01
14	C20:2n6 (cis-11,14-eicosadienoic)	29.98	0.46 ± 0.01	2.53 ± 0.01 ^a^	1.60 ± 0.01	0.66 ± 0.01	1.60 ± 0.00	1.12 ± 0.01	0.52 ± 0.04	0.48 ± 0.01	0.21 ± 0.01	0.28 ± 0.05	0.94 ± 0.04
15	C20:4n6 (arachidonic)	32.03	4.73 ± 0.02	10.79 ± 0.00	4.01 ± 0.03	3.18 ± 0.03	5.42 ± 0.02	5.83 ± 0.00	7.63 ± 0.04	16.37 ± 0.02 ^a^	2.42 ± 0.00	3.52 ± 0.08	3.04 ± 0.16
16	C20:5n3 (cis-5,8,11,14,17-eicosapentaenoic)	34.05	22.6 ± 0.00	15.6 ± 0.01	12.50 ± 0.03	31.8 ± 0.83 ^a^	15.3 ± 0.07	13.4 ± 0.05	19.2 ± 0.25	11.8 ± 0.20	12.9 ± 0.02	24.9 ± 0.02	17.9 ± 0.19
17	C22:5n3 (cis-7,10,13,16,19-docosapentaenoic)	37.81	1.63 ± 0.05	9.10 ± 0.02 ^a^	2.79 ± 0.01	0.75 ± 0.01	3.37 ± 0.02	2.02 ± 0.01	2.33 ± 0.03	9.16 ± 0.02 ^a^	0.47 ± 0.00	1.78 ± 0.01	0.88 ± 0.05
18	C22:6n3 (cis-4,7,10,13,16,19-docosahexaenoic)	38.93	16.5 ± 0.04	11.5 ± 0.01	28.4 ± 0.12	15.9 ± 0.10	16.0 ± 0.04	17.1 ± 0.01	25.8 ± 0.20	n.d.	39.6 ± 0.02 ^a^	17.9 ± 0.05	21.0 ± 1.26
	∑ SFAs		30.1 ± 0.06	26.5 ± 0.11	28.0 ± 0.22	26.4 ± 0.11	30.9 ± 0.09	24.4 ± 0.03	31.6 ± 0.69	34.8 ± 0.08	37.6 ± 0.08 ^a^	28.6 ± 0.01	26.7 ± 0.56
	∑MUFAs		18.2 ± 0.07	17.0 ± 0.06	18.1 ± 0.01	16.0 ± 0.54	19.84 ± 0.03	32.5 ± 0.01 ^a^	11.8 ± 0.13	21.6 ± 0.07	6.29 ± 0.00	13.2 ± 0.12	26.0 ± 0.21
	∑PUFAs		51.6 ± 0.01	56.5 ± 0.18	53.9 ± 0.24	57.6 ± 0.64	49.28 ± 0.06	43.0 ± 0.02	56.6 ± 0.56	43.6 ± 0.15	56.1 ± 0.08	58.1 ± 0.11 ^a^	47.3 ± 0.77
	crude lipids (% FW)		2.88 ± 0.17	2.79 ± 0.29	2.37 ± 0.18	2.75 ± 0.35	2.78 ± 0.32	4.29 ± 0.42 ^a^	1.37 ± 0.18	1.90 ± 0.14	3.32 ± 0.26	3.90 ± 0.14 ^a^	1.91 ± 0.13

Values (mean ± standard deviation) are percentages of the total fatty acids from three determinations. FAME: fatty acid methyl ester; n.d.; not detected; RT: retention time; FW: fresh weight; PUFAs: total polyunsaturated fatty acids; MUFAs: total monounsaturated fatty acids; SFAs: total saturated fatty acids; ^a^ The mean value is significantly (*p* < 0. 05) highest among the studied species. The sample numbers (S1 to S11) are correspondent to Table 1.

**Table 5:** Fat quality indices of lipids obtained from shellfish species.

Components	S1	S2	S3	S4	S5	S6	S7	S8	S9	S10	S11
PUFAs: SFAs	1.71 ± 0.00	2.13 ± 0.02	1.93 ± 0.02	2.18 ± 0.03 ^a^	1.60 ± 0.01	1.76 ± 0.00	1.79 ± 0.06	1.25 ± 0.01	1.49 ± 0.01	2.03 ± 0.00	1.77 ± 0.07
∑ n-3 PUFAs ^#^	44.8 ± 0.01	40.0 ± 0.01	47.7 ± 0.19	*51.6 ± 0.70* ^a^	41.5 ± 0.03	34.6 ± 0.04	48.0 ± 0.43	24.2 ± 0.18	53.2 ± 0.05 ^a^	53.0 ± 0.12 ^a^	41.7 ± 1.12
∑ n-6 PUFA ^#^	6.82 ± 0.02	16.5 ± 0.16	6.22 ± 0.04	5.92 ± 0.05	7.80 ± 0.03	8.39 ± 0.02	8.55 ± 0.13	19.4 ± 0.03 ^a^	2.95 ± 0.03	5.11 ± 0.01	5.65 ± 0.35
n-3/n-6 PUFA	6.58 ± 0.02	2.42 ± 0.02	7.67 ± 0.02	8.73 ± 0.20	5.32 ± 0.02	4.13 ± 0.01	5.62 ± 0.03	1.25 ± 0.01	18.05 ± 0.15 ^a^	10.4 ± 0.04	7.39 ± 0.65
AI	0.58 ± 0.02	0.41 ± 0.00	0.34 ± 0.01	0.48 ± 0.00	0.48 ± 0.01	0.33 ± 0.00	0.37 ± 0.00	0.83 ± 0.00 ^a^	0.60 ± 0.00	0.49 ± 0.00	0.37 ± 0.01
TI	0.19 ± 0.00	0.18 ± 0.00	0.16 ± 0.00	0.15 ± 0.00	0.20 ± 0.00	0.18 ± 0.00	0.18 ± 0.00	0.35 ± 0.00 ^a^	0.20 ± 0.00	0.15 ± 0.00	0.17 ± 0.01
h/H	3.03 ± 0.02	4.36 ± 0.03 ^a^	3.90 ± 0.05	3.77 ± 0.01	3.00 ± 0.01	4.41 ± 0.01 ^a^	3.42 ± 0.00	2.36 ± 0.01	1.99 ± 0.01	3.18 ± 0.00	3.66 ± 0.10

^#^ Values (mean ± standard deviation) from three determinations. PUFAs: total polyunsaturated fatty acids; MUFAs: total monounsaturated fatty acids; SFAs: total saturated fatty acids; n-3: omega-3; n-6: omega-6; h/H: ratios of hypocholesterolemic (h)/hypercholesterolemic (H) fatty acids; TI: thrombogenic index; and AI: atherogenic index. ^a^ The mean value is significantly (*p* < 0.05) highest among the studied species. The sample numbers (S1 to S11) are correspondent to Table 1.

**Figure A1:**
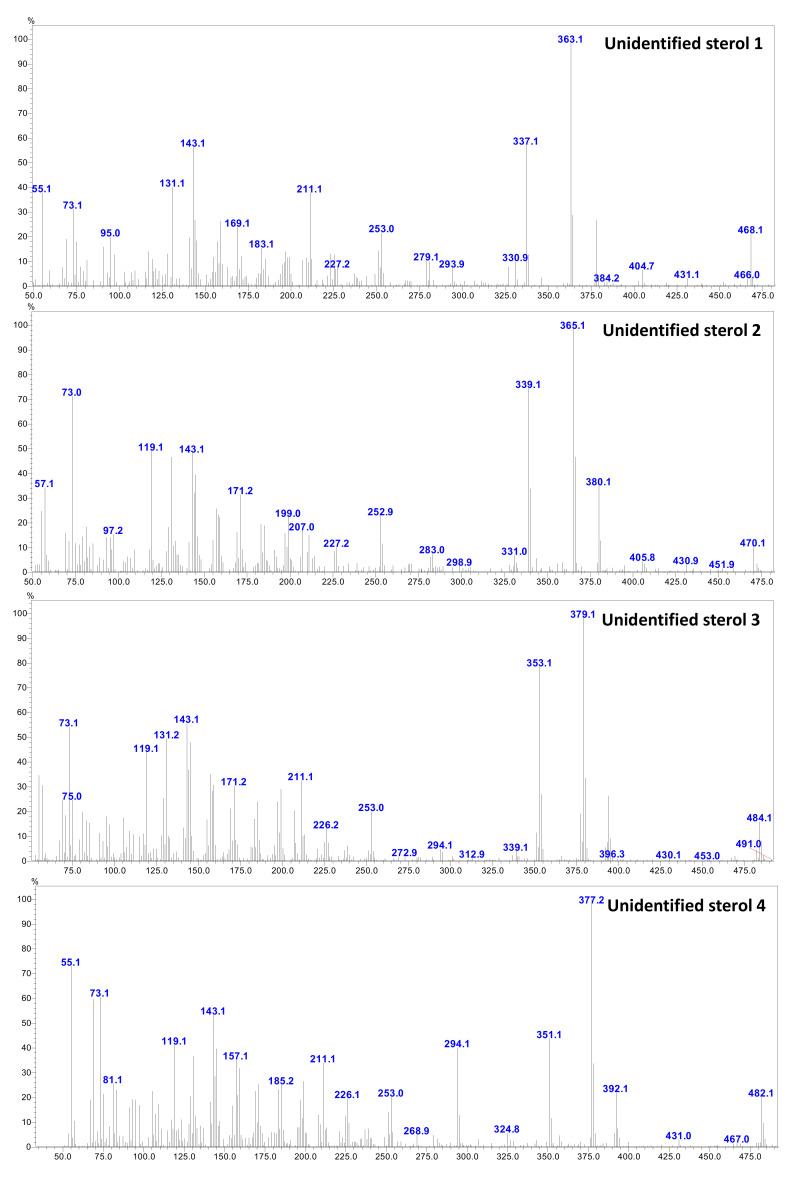
The mass spectrometric fragmentation pattern of unknown sterols observed in the present investigation.

## Data Availability

Data is contained within the article.

## Appendix A. Outline of the Method Used for Extraction of Crude Lipids from the Shellfish Species

**Extraction of crude lipids**
30 g fresh edible portions (aliquoted in falcon tubes and stored at −80 °C) were transferred to 250 mL conical flask
↓
Samples were homogenized with 150 mL isopropyl alcohol/cyclohexane (10:12, *v/v*) containing 0.075% butylated hydroxytoluene (BHT: *w/v*; antioxidant)
↓
Bath sonication (JAC-2010; 300 w, 60 Hz, for 10 min) for the efficient disintegration and complete extraction
↓
Centrifuged at 8000× *g* (10 min at 4 °C), the supernatant was collected, and pellets were extracted again with 100 mL of cyclohexane
↓
Supernatants from both extractions were pooled (total volume of ~200 mL) and partitioned with 100 mL of 1 M sodium chloride (NaCl)
↓
The upper cyclohexane phase containing crude lipids were collected, filtered over anhydrous sodium sulfate, transferred to a preweighted 250 mL round bottom flask, and vacuum-dried in a rotary evaporator at 35 °C
↓
The contents of crude lipids were determined gravimetrically and recovered in 8 mL cyclohexane/dichloromethane (DCM) (3:1, *v/v*) containing 0.1% BHT and utilized as follows:
(a) One mL of the crude lipids sample was filtered through a 0.45 μm PTFE syringe filter and transferred to a 1.5 mL autosampler vial for tocols analysis by utilizing HPLC-DAD. (b) 500 µL of the sample was converted to FAMEs and analyzed by GC- FID and GC-MS. (c) 200 µL of the sample was hydrolyzed, silylated, and utilized to quantify the sterols using GC-MS. (d) Sample containing 20 mg of crude lipids were transferred to a 5 mL glass, vacuum-dried in a rotary evaporator at 35 °C, lipids recovered in 1 mL dimethyl sulfoxide DMSO, and utilized for cytotoxicity studies.

## Appendix B. Outline of Methods Used for the Hydrolysis and Silylation of Sterols for GC-MS Analysis (A) and the Preparation of Fatty Acid Methyl Esters (B)

**(A) Hydrolysis and silylation of sterols for GC-MS analysis**
200 µL of crude lipids sample and 50 µg of 5β-cholestan-3α-ol (internal standard dissolved in dichloromethane) were transferred into a 5 mL glass vial fitted with a Teflon-lined screw cap, and contents were evaporated to dryness using a rotary evaporator at 35 °C
↓
1 mL of 0.5 M methanolic potassium hydroxide (KOH) were added and placed in a water bath at 85 °C for 15 min (for hydrolysis)
↓
Hydrolyzed samples were immediately cooled in ice and partitioned with 2 mL of cyclohexane and 1 mL of 1 M sodium chloride (NaCl)
↓
One mL from the upper cyclohexane phase containing sterols was carefully transferred into a new 5 mL Teflon lined glass tube and vacuum-dried in a rotary evaporator at 35 °C
↓
For silylation of sterols, 1 mL pyridine, and 50 µL of N,O-bis(trimethylsilyl)trifluoroacetamide (BSTFA) containing 1% trimethylchlorosilane (TMCS) and added and incubated at 60 °C for 60 min
↓
After incubation, contents were cooled in ice, filtered through a 0.45 μm PTFE syringe filtered and transferred to a 1.5 mL autosampler vial for gas chromatograph-mass spectrometry (GC-MS) analysis
**(B) Preparation of fatty acid methyl esters (FAMEs)**
500 µL of crude lipids sample was transferred into a 5 mL glass vial fitted with a Teflon-lined screw cap
↓
Contents were evaporated to dryness using a rotary evaporator at 35 °C, and 1 mL of boron trifluoride-methanol solution (14% in methanol), and incubated for 10 min at 60 ° in a heat block
↓
After cooling, the FAMEs were washed with 1 M NaCl and recovered in 2 mL hexane. A pinch of anhydrous sodium sulfate (Na_2_SO_4_) was added to the recovered sample (hexane) to absorb the traces of water
↓
One mL of sample was filtered through a 0.45 μm PTFE syringe filter and transferred to a 1.5 mL autosampler vial for GC-FID analysis and GC-MS analysis
